# Validation of a method for identifying nursing home admissions using administrative claims

**DOI:** 10.1186/1472-6963-7-202

**Published:** 2007-12-10

**Authors:** Ilene H Zuckerman, Masayo Sato, Van Doren Hsu, Jose J Hernandez

**Affiliations:** 1 Department of Pharmaceutical Health Services Research, University of Maryland School of Pharmacy, Baltimore, Maryland, USA; 2 Department of Pharmacy Practice, University of Puerto Rico School of Pharmacy, San Juan, Puerto Rico

## Abstract

**Background:**

Currently there is no standard algorithm to identify whether a subject is residing in a nursing home from administrative claims. Our objective was to develop and validate an algorithm that identifies nursing home admissions at the resident-month level using the MarketScan Medicare Supplemental and Coordination of Benefit (COB) database.

**Methods:**

The computer algorithms for identifying nursing home admissions were created by using provider type, place of service, and procedure codes from the 2000 – 2002 MarketScan Medicare COB database. After the algorithms were reviewed and refined, they were compared with a detailed claims review by an expert reviewer. A random sample of 150 subjects from the claims was selected and used for the validity analysis of the algorithms. Contingency table analysis, comparison of mean differences, correlations, and t-test analyses were performed. Percentage agreement, sensitivity, specificity, and Kappa statistics were analyzed.

**Results:**

The computer algorithm showed strong agreement with the expert review (99.9%) for identification of the first month of nursing home residence, with high sensitivity (96.7%), specificity (100%) and a Kappa statistic of 0.97. Weighted Pearson correlation coefficient between the algorithm and the expert review was 0.97 (*p *< 0.0001).

**Conclusion:**

A reliable algorithm indicating evidence of nursing home admission was developed and validated from administrative claims data. Our algorithm can be a useful tool to identify patient transitions from and to nursing homes, as well as to screen and monitor for factors associated with nursing home admission and nursing home discharge.

## Background

Administrative claims databases have been utilized frequently in health services research [1-4]. For instance, automated claims data protocols are used to identify chronic conditions, predict future health care costs, screen for population at risk for outcomes, and review the appropriateness of medical procedures (including surgery and hospital admission) and drug prescribing [5-7]. Many studies have assessed the validity and reliability of these protocols for a number of clinical conditions [8-13]. Although claims databases have limitations such as lack of information on important risk factors (i.e., smoking status and family histories), they remain a good resource for a number of reasons. The use of large administrative databases is relatively inexpensive, minimizes selection bias, and increases external validity compared to the primarily collected dataset. Automated claims data contain more accurate and complete information on variables related to payment. Since they are rich in patient utilization variables, and since patient utilization variables can be linked to the patient health care environment, large administrative databases can be used to identify nursing home related transitions [14-18]. However, claims data have unique limitations that may diminish the validity of results [9,10,19,20]. In addition, the sensitivity of claims data to capture and assess case mix has been a key issue [2,21].

Nursing home care is the most expensive type of long-term care [22]. Poor health outcomes, including death, have been found to be associated with nursing home placement [23,24]. A recent meta-analysis using data that is nationally representative of the U.S. population found that three or more activities of daily living dependencies, cognitive impairment, and prior nursing home use were the strongest predictors of nursing home admissions [25]. It is extremely important to screen and monitor for factors associated with nursing home admissions. However, no standard algorithm to identify nursing home admissions has been developed, making it difficult to isolate true admissions. Utilization of administrative claims databases for nursing home research has not been extensively explored. There is no direct way to identify whether a subject is in a nursing home from administrative claims [2,16,26-28]. For example, if a subject's health insurance does not cover the nursing home care (i.e., the nursing home charges), then there will not be a claim for the charges. However, if a subject is in a nursing home under that circumstance, there will be other covered charges, for example, physician visits, during the nursing home stay. These other covered charges may be captured, albeit indirectly, in the claims by examination of the place of service and procedure codes. Beusterien et al. investigated the impact of rivastigmine use on the risk of nursing home placement using the MarketScan database [29]. Nursing home placement was identified by a record of a nursing home claim on two separate dates. However, it is unclear whether their methodology included only claims submitted by nursing home providers or claims that indicated nursing home stays through place of service and procedure codes. Hence, it is not simple to identify nursing home admission from claims because nursing home stays are covered fully, partially, or not at all by various payers, unless one has access to *all *sources of payment, including self-payment.

Awareness of the potential pitfalls for identifying nursing home admission in the use of large claims data can help prevent misclassification and improve the validity and efficiency of analysis. Therefore, the development of a valid and reliable algorithm to identify nursing home admissions is imperative. The algorithm may help to screen high risk elderly or monitor elderly in terms of nursing home admission. The objectives of this study are to develop an algorithm that identifies nursing home stays on a month level and to validate the algorithm against a "gold standard" measure of monthly nursing home stays using the MarketScan Medicare Supplemental and Coordination of Benefit database.

## Methods

### Study subjects and data sources

Data were obtained from the 2000–2002 MarketScan Medicare Supplemental and Coordination of Benefit database. The database, produced by Thomson Medstat, contains information on a convenience sample of privately insured Medicare-eligible retirees who are covered by employer-sponsored Medicare supplemental benefit plans [30]. The database includes all employer and Medicare coordination of benefits for those enrollees who have both employer coverage and Medicare coverage. The longitudinal database links medical and prescription drug administrative claims with person-level enrollment data. All MarketScan Medicare inpatient and outpatient service claims containing information on any of the following variables were extracted: (1) provider type suggestive of a nursing home (e.g., long-term care facility); (2) place of service suggestive of a nursing home (e.g., skilled nursing facility); or (3) CPT (Current Procedure Terminology) procedure code suggestive of care specific to a nursing home. Table 1 includes a description of these claims codes.

**Table 1:** Claims codes screened for nursing home admission incidents

**Code**	**Description**
**Provider type codes**	
30	Long term care facility
31	Extended care facility
32	Geriatric hospital
33	Convalescent care facility
34	Intermediate care facility
**Place of service codes**	
27	Inpatient long term care
31	Skilled nursing facility
32	Nursing facility
33	Custodial care facility
**Procedure codes**	
99301	Evaluation and management of a new or established patient involving an annual nursing facility assessment: 30 minutes at the bedside.
99302	Evaluation and management of a new or established patient involving an annual nursing facility assessment of a complication or a new problem: 40 minutes at the bedside.
99303	Evaluation and management of a new or established patient involving an annual nursing facility assessment at the time of initial admission to the facility: 50 minutes at the bedside.
99311	Subsequent nursing facility care, per day, for the evaluation and management of a new or established patient: 15 minutes at the bedside.
99312	Subsequent nursing facility care, per day, for the evaluation and management of a new or established patient who is responding inadequately to therapy or has developed a minor complication: 25 minutes at the bedside.
99313	Subsequent nursing facility care, per day, for the evaluation and management of a new or established patient who has developed a significant complication or a new problem: 35 minutes at the bedside.
99315	Nursing facility discharge day management; 30 minutes or less
99316	Nursing facility discharge day management; more than 30 minutes
99379	Physician supervision of a nursing facility patient (patient not present) requiring complex and multidisciplinary care; 15–29 minutes
99380	Physician supervision of a nursing facility patient (patient not present) requiring complex and multidisciplinary care modalities; 30 minutes or more
G0066	Physician supervision of a nursing facility patient (patient not present); 30 minutes or more per month
99199	Unlisted special service, procedure or report
99321	Domiciliary or rest home visit for the evaluation and management of a new patient; the presenting problems are of low severity
99322	Domiciliary or rest home visit for the evaluation and management of a new patient; the presenting problems are of moderate severity
99323	Domiciliary or rest home visit for the evaluation and management of a new patient; the presenting problems are of high complexity
99331	Domiciliary or rest home visit for the evaluation and management of a new patient; the patient is stable, recovering or improving
99332	Domiciliary or rest home visit for the evaluation and management of a new patient; the patient is responding inadequately to therapy or has developed a minor complication
99333	Domiciliary or rest home visit for the evaluation and management of a new patient; the patient is unstable or has developed a significant complication or a significant new problem

### Creating the computer algorithms for identifying nursing home stays

Claims were sorted by person identifier and service dates, and from this an event day-level file was created. For each person-day, a variable (RULE) was assigned that indicates the type of nursing home (NH)-related claims identified for that date. The value of RULE was specific to the provider [1 = NH-related or 0 = not], place of service [1 = NH-related or 0 = not], procedure [1 = NH-related or 0 = not] and source of claim (inpatient or outpatient claim). Thus the value for RULE was a string of 6 characters of 0s and 1s: the 1^st ^character is a flag of a claim with a NH provider type from an inpatient claim; the 2^nd ^character is a flag of a claim with a NH provider type from an outpatient claim; the 3^rd ^character is a flag of a claim with a NH place of service from an inpatient claim; the 4^th ^character is a flag of a claim with a NH place of service from an outpatient claim; the 5^th ^character is a flag of a claim with a NH procedure code from an inpatient claim; the 6^th ^character is a flag of a claim with a NH procedure code from an outpatient claim.

For example, RULE = '100000' means that for a specific person-day there was one or more inpatient claims that had a NH provider type. Furthermore, on that specific date, there were no inpatient or outpatient claims with a NH place of service or NH procedure code. RULE = '101000'means that for a specific person-day there was one or more inpatient claims that had a NH provider type and an inpatient claim with a NH place of service, and RULE = '111111' means that for a specific person-day there was one or more inpatient and outpatient claims that had a NH provider type, a NH place of service, and a NH procedure code. There were 833,669 person-days where the provider type, place of service or procedure code was indicative of a nursing home stay. These person-days represented 90,465 subjects. The frequency distribution of the claims codes RULE variable is displayed in Table 2.

**Table 2:** Frequencies of nursing home evidence types among days with any evidence (n = 833,669 person-days)

**RULE**	**Description**	**Frequency**	**Percent**
000101	outpatient POS and outpatient PROC	448,959	53.85
000001	outpatient PROC only	143,515	17.21
000100	outpatient POS only	137,355	16.48
010100	outpatient PROVIDER and outpatient POS	43,413	5.21
000010	inpatient PROC only	19,213	2.30
001010	inpatient POS and inpatient PROC	12,152	1.46
010000	outpatient PROVIDER only	10,444	1.25
101000	inpatient PROVIDER and inpatient POS	4,830	0.58
010101	outpatient PROVIDER and outpatient POS and outpatient PROC	4,513	0.54
001000	inpatient POS only	3,844	0.46
100000	inpatient PROVIDER only	3,359	0.40
101010	inpatient PROVIDER and inpatient POS and inpatient PROC	564	0.07
010001	outpatient PROVIDER and outpatient PROC	351	0.04
001111	inpatient POS and outpatient POS and inpatient PROC and outpatient PROC	232	0.03
100010	inpatient PROVIDER and inpatient PROC	172	0.02
111100	inpatient PROVIDER and outpatient PROVIDER and inpatient POS and outpatient POS	107	0.01
001100	inpatient POS and outpatient POS	98	0.01
001001	inpatient POS and outpatient PROC	78	0.01
000011	inpatient and outpatient PROC only	72	0.01
001011	inpatient POS and inpatient PROC and outpatient PROC	62	0.01
000111	outpatient POS and inpatient PROC and outpatient PROC	58	0.01
101001	inpatient PROVIDER and inpatient POS and outpatient PROC	50	0.01
100101	inpatient PROVIDER and outpatient POS and outpatient PROC	45	0.01
110000	inpatient PROVIDER and outpatient PROVIDER	40	< 0.01
110100	inpatient PROVIDER and outpatient PROVIDER and outpatient POS	39	< 0.01
111000	inpatient PROVIDER and outpatient PROVIDER and inpatient POS	31	< 0.01
010110	outpatient PROVIDER and outpatient POS and inpatient PROC	17	< 0.01
100100	inpatient PROVIDER and outpatient POS	15	< 0.01
100001	inpatient PROVIDER and outpatient PROC	11	< 0.01
111111	inpatient PROVIDER and outpatient PROVIDER and inpatient POS and outpatient POS and inpatient PROC and outpatient PROC	5	< 0.01
011010	outpatient PROVIDER and inpatient POS and inpatient PROC	4	< 0.01
111010	inpatient PROVIDER and outpatient PROVIDER and inpatient POS and inpatient PROC	4	< 0.01
000110	outpatient POS and inpatient PROC	3	< 0.01
010010	outpatient PROVIDER and inpatient PROC	3	< 0.01
110101	inpatient PROVIDER and outpatient PROVIDER and outpatient POS and outpatient PROC	3	< 0.01
011111	outpatient PROVIDER and inpatient POS and outpatient POS and inpatient PROC and outpatient PROC	2	< 0.01
100011	inpatient PROVIDER and inpatient PROC and outpatient PROC	2	< 0.01
101011	inpatient PROVIDER and inpatient POS and inpatient PROC and outpatient PROC	2	< 0.01
001101	inpatient POS and outpatient POS and outpatient PROC	1	< 0.01
111101	inpatient PROVIDER and outpatient PROVIDER and inpatient POS and outpatient POS and outpatient PROC	1	< 0.01

POS = place of service; PROC = procedure code

Each RULE type was categorized as "PROBABLE," "POSSIBLE," or "UNLIKELY" for its rating of nursing home evidence. Strong evidence of a nursing home stay was coded as "PROBABLE" (e.g., one or more outpatient claims that had a NH place of service and an outpatient claim with a NH procedure code: RULE = 000101); ambiguous evidence of a nursing home stay was coded as "POSSIBLE" (e.g., one or more outpatient claims that had only NH procedure code: RULE = 000001); and "UNLIKELY" (e.g., one or more inpatient claims that had only NH procedure code: RULE = 000010) indicated no evidence of a nursing home stay. Table 3 contains a full description of the protocol used to classify each RULE as PROBABLE, POSSIBLE, or UNLIKELY. Two computerized algorithms were developed to assign a level of evidence for a nursing home stay for each person-month. Algorithm 1 defined a person-month as a nursing home stay if there was at least one person-day in the month where the nursing home RULE = "PROBABLE" was present; otherwise the person-month was considered not to be a nursing home stay. Algorithm 2 defined a person-month as a nursing home stay if there was at least one person-day in the month where the nursing home RULE = "PROBABLE" or RULE = "POSSIBLE" was present; otherwise the person-month was considered not to be a nursing home stay.

**Table 3:** Modified nursing home rules, based on preliminary expert review

**RULE**	**Description**	**NH status PROBABLE, POSSIBLE, or UNLIKELY,**
000001	outpatient PROC only	POSSIBLE
000010	inpatient PROC only	UNLIKELY
000011	inpatient and outpatient PROC only	POSSIBLE
000100	outpatient POS and SPECIAL SERVICE or REST HOME PROC	PROBABLE*
	outpatient POS only	UNLIKELY*
000101	outpatient POS and outpatient PROC	PROBABLE
000110	outpatient POS and inpatient PROC	PROBABLE
000111	outpatient POS and inpatient PROC and outpatient PROC	PROBABLE
001000	inpatient POS and REST HOME PROC	POSSIBLE^†^
	inpatient POS and SPECIAL SERVICE PROC	PROBABLE^†^
	inpatient POS only	UNLIKELY^†^
001001	inpatient POS and outpatient PROC	PROBABLE
001010	inpatient POS and inpatient PROC	PROBABLE
001011	inpatient POS and inpatient PROC and outpatient PROC	PROBABLE
001100	inpatient POS and outpatient POS	PROBABLE
001101	inpatient POS and outpatient POS and outpatient PROC	PROBABLE
001111	inpatient POS and outpatient POS and inpatient PROC and outpatient PROC	PROBABLE
010000	outpatient PROVIDER only	UNLIKELY
010001	outpatient PROVIDER and outpatient PROC	POSSIBLE
010010	outpatient PROVIDER and inpatient PROC	PROBABLE
010100	outpatient PROVIDER and outpatient POS	PROBABLE
010101	outpatient PROVIDER and outpatient POS and outpatient PROC	PROBABLE
010110	outpatient PROVIDER and outpatient POS and inpatient PROC	PROBABLE
011010	outpatient PROVIDER and inpatient POS and inpatient PROC	PROBABLE
011111	outpatient PROVIDER and inpatient POS and outpatient POS and inpatient PROC and outpatient PROC	PROBABLE
100000	inpatient PROVIDER only	PROBABLE
100001	inpatient PROVIDER and outpatient PROC	PROBABLE
100010	inpatient PROVIDER and inpatient PROC	PROBABLE
100011	inpatient PROVIDER and inpatient PROC and outpatient PROC	PROBABLE
100100	inpatient PROVIDER and outpatient POS	PROBABLE
100101	inpatient PROVIDER and outpatient POS and outpatient PROC	PROBABLE
101000	inpatient PROVIDER and inpatient POS	PROBABLE
101001	inpatient PROVIDER and inpatient POS and outpatient PROC	PROBABLE
101010	inpatient PROVIDER and inpatient POS and inpatient PROC	PROBABLE
101011	inpatient PROVIDER and inpatient POS and inpatient PROC and outpatient PROC	PROBABLE
110000	inpatient PROVIDER and outpatient PROVIDER	PROBABLE
110100	inpatient PROVIDER and outpatient PROVIDER and outpatient POS	PROBABLE
110101	inpatient PROVIDER and outpatient PROVIDER and outpatient POS and outpatient PROC	PROBABLE
111000	inpatient PROVIDER and outpatient PROVIDER and inpatient POS	PROBABLE
111010	inpatient PROVIDER and outpatient PROVIDER and inpatient POS and inpatient PROC	PROBABLE
111100	inpatient PROVIDER and outpatient PROVIDER and inpatient POS and outpatient POS	PROBABLE
111101	inpatient PROVIDER and outpatient PROVIDER and inpatient POS and outpatient POS and outpatient PROC	PROBABLE
111111	inpatient PROVIDER and outpatient PROVIDER and inpatient POS and outpatient POS and inpatient PROC and outpatient PROC	PROBABLE

POS = place of service; PROC = procedure code

*If claim had one of the following procedure codes; 99199, 99321, 99322, 99323, 99331, 99332, 99333, it was classified on "PROBABLE" otherwise it was classified on "UNLIKELY".

^† ^If claim had one of the following procedure codes; 99321, 99322, 99323, 99331, 99332, 99333, it was classified on "POSSIBLE". If claim had 99199, then it was classified on "PROBABLE" otherwise "UNLIKELY".

### Validating the algorithms against a "gold standard"

A random sample stratified by nursing home evidence for their first nursing home month of 150 subjects was selected: 50 subjects with evidence = "PROBABLE"; 50 subjects with evidence = "POSSIBLE," and 50 subjects with no evidence of nursing home residence during any month (= "UNLIKELY"). We used this stratification to assure that our sample contained subjects that represented a broad spectrum of our RULEs for identifying nursing home stays.

For each person in the randomly selected validation sample, all of their inpatient and outpatient administrative service claims for years 2000, 2001, and 2002 were extracted. These claims were arranged into a profile for each person that contained a line listing of the claims, sorted by service date with the following information: patient identifier, source (inpatient or outpatient), date, provider type, place of service, procedure. An expert claims reviewer reviewed the profiles. The expert reviewer was a clinical pharmacist who had over eight years of experience doing research with administrative claims and other secondary datasets. One of her areas of expertise is in developing algorithms and operationalizing definitions of specific outcomes and covariates from administrative claims. The reviewer was blinded to the computer algorithm's values. The expert reviewer recorded her assessment on a data collection sheet. For each person-month, the reviewer recorded a "1" in the box for each month determined to have any evidence of nursing home residence, and left the box blank if there was no evidence of nursing home residence. Results of the expert reviewer were entered into a database and compared to the results of the two computer algorithms. This in-depth review of claims was considered the "gold standard" for the purpose of validation of the computer-based algorithm.

Since the sampling unit was the subject, all analyses were performed with the subject as the unit of analysis. To estimate statistical measures for the entire population of interest, each subject's measures were weighted. Each person was assigned a weight which was derived from the reciprocal of the probability of selection, based upon the stratified sampling design. All results are reported using weighted measures; unweighted measures are also included for measures of agreement, sensitivity, specificity and Kappa.

To compare the computer algorithms to the "gold standard" assessment of nursing home residence at the month level, a month from each sample subject was randomly selected to report percent agreement, sensitivity, specificity and predictive values. In addition, the Kappa statistic is reported. The Kappa statistic determines the extent of agreement between two or more measures beyond what would be expected by chance. The standard error of Kappa was used to generate 95 percent confidence intervals [31]. Previously established guidelines were used to interpret the Kappa statistics. A Kappa greater than 0.75 indicates excellent agreement, values between 0.4 and 0.75 indicate fair to good agreement, and values less than 0.4 indicate poor agreement [32]. *A priori *acceptable values for agreement, sensitivity and specificity were set at greater than 0.8, and *a priori *acceptable values for Kappa were set at greater than 0.75. Correlations and paired t-tests were used to compare the two methods' total number of nursing home months identified per subject.

## Results

The population comprises 520,260 subjects, represented by the stratified sample of 150 subjects (Table 4). Mean age of the sample population was 74 years old; 55 percent were female. Table 5 compares the "gold standard" review to the computer algorithms. Both algorithms had high agreement and specificity. However, Algorithm 2, a broader definition including ambiguous nursing home evidence, had higher sensitivity than Algorithm 1. Algorithm 2 also had a higher Kappa statistic, indicating that this algorithm had higher chance-corrected agreement with the "gold standard" and was less susceptible to chance agreement. Algorithm 1 did not meet the *a priori *criteria for acceptable sensitivity or for Kappa in either the unweighted or weighted measures. Positive predictive values were high for both algorithms (1.00 and 0.97 for Algorithm 1 and Algorithm 2, respectively) and negative predictive values were above 0.99 for both algorithms. Algorithm 1 agreed with the expert reviewer to the exact month in 97.9 percent of the subjects, and was accurate within two months in 98.0 percent of subjects. Algorithm 2 performed slightly better, with exact month agreement in 99.1 percent subjects, and within two months in 99.3 percent of subjects. Detailed information on distribution of the expert reviewer's responses and algorithm determinations is shown in Table 6. The total number of nursing home residence months per subjects during the study period was similar among all three measures, with a mean difference of less than one month for each algorithm, when compared to the expert review (Table 7). The high correlations between each algorithm and the expert review also suggest agreement on this measure, although Algorithm 2's correlation coefficient was slightly higher than Algorithm 1 (r = 0.97 vs. 0.83, respectively). Algorithm 2 is more likely to have higher validity.

**Table 4:** Validation sample stratification and weights

**Stratification group**	**Validation sample size (n = 150)**	**Study population size (n = 520,260)**	**Probability of selection**	**Weight**
First nursing home residence month with "PROBABLE" evidence	50	27,668	0.001807	553.36
First nursing home residence month with "POSSIBLE" evidence	50	8,970	0.005574	179.40
All months with "UNLIKELY" evidence	50	483,622	0.000103	9,672.44

**Table 5:** Frequency and agreement between methods of determination of nursing home residence (n = 150, Weighted n = 520,260)

	Algorithm 1*	**Algorithm 2**^†^	**Expert Review**
Frequency (%) of NH stay			
Unweighted	6 (4.0)	18 (12.0)	18 (12.0)
Weighted	3,320 (0.6)	5,473 (1.1)	5,473 (1.1)
Correspondence with expert review			
Agreement			
Unweighted	92.0%	98.7%	-
Weighted	99.6%	99.9%	-
Sensitivity			
Unweighted	0.33	0.94	-
Weighted	0.61	0.97	-
Specificity			
Unweighted	1.00	0.99	-
Weighted	1.00	1.00	-
Kappa (95% CI)			
Unweighted	0.47 (0.22, 0.71)	0.93 (0.85, 1.00)	-
Weighted	0.75 (0.74, 0.76)	0.97 (0.96, 0.97)	-

NH = nursing home; CI = confidence interval

*Algorithm 1 defined a person-month as a nursing home stay if there was at least one day in the month where the nursing home RULE = "PROBABLE"; otherwise the person-month was considered not to be a nursing home stay.

† Algorithm 2 defined a person-month as a nursing home stay if there was at least one day in the month where the nursing home RULE = "PROBABLE" or RULE = "POSSIBLE"; otherwise the person-month was considered not to be a nursing home stay.

**Table 6:** Unweighted frequency between methods of determination of nursing home residence (n = 150)

		**Frequency (%)**		
		
		**Expert Review**		
		
		**NH**	**Non-NH**	**Total**
		
Algorithm 1*	**NH**	6 (4.0)	0 (0.0)	6 (4.0)
	**Non-NH**	12 (8.0)	132 (88.0)	144 (96.0)
		18 (12.0)	132 (88.0)	150 (100)
**Algorithm 2**^†^	**NH**	17 (11.3)	1 (0.7)	18 (12.0)
	**Non-NH**	1 (0.7)	131 (87.3)	132 (88.0)
		18 (12.0)	132 (88.0)	150 (100)

NH: Evidence of nursing home residence

*Algorithm 1 defined a person-month as a nursing home stay if there was at least one day in the month where the nursing home

RULE = "PROBABLE"; otherwise the person-month was considered not to be a nursing home stay.

^†^Algorithm 2 defined a person-month as a nursing home stay if there was at least one day in the month where the nursing home

RULE = "PROBABLE" or RULE = "POSSIBLE"; otherwise the person-month was considered not to be a nursing home stay.

**Table 7:** Comparison of total count of nursing home residence months(n = 150, Weighted n = 520,260)

	**Weighted mean difference in total number of nursing home residence months (95% CI)**	**p-value**	**Weighted Pearson correlation coefficient**	**p-value**
	
Algorithm 1*vs. Expert Review	-0.13 (-0.36, 0.11)	0.30	0.83	<0.0001
**Algorithm 2**^†^**vs. Expert Review**	-0.01 (-0.11, 0.10)	0.93	0.97	<0.0001

CI = confidence interval

*Algorithm 1 defined a person-month as a nursing home stay if there was at least one day in the month where the nursing home RULE = "PROBABLE"; otherwise the person-month was considered not to be a nursing home stay.

^†^Algorithm 2 defined a person-month as a nursing home stay if there was at least one day in the month where the nursing home RULE = "PROBABLE" or RULE = "POSSIBLE"; otherwise the person-month was considered not to be a nursing home stay.

## Discussion

A reliable algorithm indicating evidence of nursing home admission was developed and validated from administrative claims data. Algorithm 2 met minimal *a priori *criteria for sensitivity, specificity and agreement, suggesting that the algorithm is a valid measure of nursing home residence. Although Algorithm 1 performed well with regard to measuring the total number of nursing home residence months, its sensitivity reached only 61 percent (weighted) with a Kappa statistic suggesting fair to good agreement. Based on a Kappa statistic of 0.97, Algorithm 2 displayed excellent agreement and higher validity. Thus, "relaxing" the definition for a nursing home stay by including those rules with a "POSSIBLE" evidence rating improved sensitivity without sacrificing specificity.

The methodology compares algorithms based on nursing home-related claims for identifying nursing home stays to a "gold standard" measure, which is an expert's review of all claims (nursing-home related and non-nursing home-related claims). One limitation is that the "gold standard" is not an actual observation of whether or not the subject is in the nursing home; it is also based on administrative claims analysis. However, the "gold standard" is more robust in that it includes a review of all administrative claims (inpatient and outpatient) and it is an implicit review by an expert who has research experience with claims analysis, and specifically with nursing home claims. Having two or more experts review the claims and adjudicating discrepancies would strengthen the "gold standard" measure, but resources precluded the use of more than one reviewer.

Since our algorithms were developed and validated using the MarketScan Medicare Supplemental and Coordination of Benefit database, they may not be applicable to other claims databases. The MarketScan database has unique characteristics. It is a collection of coordination of benefits claims, which means that if a service claim was paid completely by Medicare, it may not be in the Coordination of Benefit database. This scenario is likely to happen with Medicare qualified skilled nursing facility stays since Medicare covers full cost for the first 20 days. In addition, the subjects in this study cohort may be different from the general elderly Medicare population. They are, on average, likely to be younger and have better income, education, and health [5].

Three different indicators were used to identify nursing home in the claims databases in this study: provider type, place of service, and procedure code. Since claims data primarily serve billing purposes, provider type and procedure codes are likely to be more accurate than place of service. Place of service codes do not directly affect reimbursement. Also, it is difficult for both the computer algorithm and the expert reviewer to differentiate between nursing homes and assisted living facilities.

The development of algorithms for identifying nursing home admission can help to reduce misclassification. Sufficient magnitude of this measurement error decreases the validity of a study's findings. Some studies using claims databases describe that their measures of nursing home admission are defined as patient admission to nursing home or long-term care facility [15,17,33], with no methodological details about how admission to nursing facilities was identified, even though most claims databases have the same measurement issues as the MarketScan database. Unless the measurement methodology is explained, it is difficult to assess how measurement errors may affect the study results. Our findings suggest that using one indicator alone on claims may miss capturing some patients with events of nursing home admission, reducing the measure's sensitivity. Researchers using claims should know characteristics of databases and be cautious about the potential pitfalls.

## Conclusion

Based upon our analysis, Algorithm 2 is a valid measure of nursing home residence when compared to a "gold standard" expert review. Using similar methodology, algorithms can be developed and applied to various administrative databases as a useful tool for screening and monitoring high risk patients for nursing home admission. Since administrative claims databases can provide large, representative samples of longitudinal patient profiles, they can be effectively used to analyze factors associated with nursing home admission.

## Competing interests

The author(s) declare that they have no competing interests.

## Authors' contributions

IHZ planned and constructed the study. IHZ and VDH carried out creation of algorithm and claims data review. IHZ, MS, and JJH interpreted data as a team and drafted the manuscript. All authors contributed to the development of this manuscript. All authors read and approved the final manuscript.

## Pre-publication history

The pre-publication history for this paper can be accessed here:


